# Comparison of ocular biometric measurements between a new swept-source optical coherence tomography and a common optical low coherence reflectometry

**DOI:** 10.1038/s41598-017-02463-z

**Published:** 2017-05-30

**Authors:** Rongrong Gao, Hao Chen, Giacomo Savini, Yaxin Miao, Xiaorui Wang, Jing Yang, Weiqi Zhao, Qinmei Wang, Jinhai Huang

**Affiliations:** 1 School of Ophthalmology and Eye Hospital, Wenzhou Medical University, Wenzhou, Zhejiang China; 2 Key Laboratory of Vision Science, Ministry of Health P.R. China, Wenzhou, Zhejiang China; 3 G.B. Bietti Foundation IRCCS, Rome, Italy

## Abstract

The purpose of the current study was to compare the measurements between a new optical biometer based on swept-source optical coherence tomography (SS-OCT), the OA-2000 (Tomey, Japan), and an optical biometer based on optical low coherence reflectometry (OLCR), the Lenstar (Haag-Streit, Switzerland). Ninety-nine eyes of 99 healthy subjects were included. The axial length (AL), central corneal thickness (CCT), anterior chamber depth (ACD), aqueous depth (AD), lens thickness (LT), keratometry (K) readings, including flat K (Kf), steep K (Ks), mean K (Km), astigmatism vectors J_0_, J_45_ at diameters of 2.5 and 3.0 mm, and white-to-white diameter (WTW) were measured three times each using both biometer in normal eyes by random sequence. Bland-Altman analysis showed good agreement between the SS-OCT and OLCR devices for AL, AD, ACD, LT, with narrow 95% LoA (−0.05 to 0.07 mm, −0.09 to 0.10 mm, −0.10 to 0.09 mm, and −0.06 to 0.22 mm, respectively), and the *P* values of ACD were both >0.05. The CCT, Kf, Ks, Km, J_0_, J_45_ and WTW values provided by the OA-2000 were in good agreement with the Lenstar, and statistically significant differences were detected for some of them but not clinical differences. The agreement was excellent especially for AL.

## Introduction

Accurate and precise measurements of ocular biometric parameters are crucial for many clinical and research applications in ophthalmology. Axial length (AL) is a fundamental parameter for calculating the most suitable intraocular lens (IOL) in cataract surgery and refractive lens exchange. A previous study reported that AL reading plays an important role in predicting postoperative refractive error and attributes to 54% of the actual refractive error^1^. Accurate measurements of anterior chamber depth (ACD), aqueous depth (AD) and keratometry play a major role in the calculation of standard as well as phakic IOLs^2, 3^. ACD is also used to screen the primary angle closure glaucoma and monitor the changes of the anterior eye segment during accommodation^4–6^. Central corneal thickness (CCT) is used to screen candidates for refractive surgery, in order to reduce the risk of postoperative ectasia^7^. CCT readings are also crucial to detect contact lens-induced edema, and help to diagnose corneal diseases and glaucoma^8–11^.

Ultrasound biometry has been the common technique for a long time to measure ACD, lens thickness (LT) and AL because of its cost-effectiveness and ease of use^8, 9^. Nevertheless, it has some shortcomings, such as corneal-probe contact with possible corneal epithelial lesions, the risk of infections and the need for topical anesthesia^12^. Since the advent of the IOLMaster (Carl Zeiss Meditec, Jena, Germany), optical biometry has been widely used and regarded as the gold standard for ocular biometric parameters^13, 14^. The Lenstar (Haag-Streit, Bem, Switzerland), which is based on optical low coherence reflectometry (OLCR), was the first competitor of the IOLMaster. A single non-contact measurement can simultaneously provide nine biometric parameters, including AL, ACD, CCT, LT, keratometry, retinal thickness, white-to-white distance (WTW) and eccentricity of the visual optical line^15^. Previous studies have demonstrated excellent intraoperator repeatability, interoperator reproducibility and accuracy of the Lenstar^16–18^. The OA-2000 (Tomey, Japan) is a newly introduced optical biometer, based on swept-source optical coherence tomography (SS-OCT). Its measurements were shown to be repeatable^19, 20^. A previous study used a functional prototype which based on OLCR might have provided different results^19^. However, to the best of our knowledge, comparison of ocular biometric parameters between the commercially available SS-OCT version of the OA-2000 and the commonly used biometer Lenstar has never been performed.

The purpose of this study was to prospectively evaluate agreement between the AL, ACD, AD, keratometry, CCT, LT and WTW measurements derived from the OA-2000 based SS-OCT and the Lenstar in normal eyes for the first time in normal eyes.

## Results

Ninety-nine right eyes of 99 healthy volunteers (58 women), with a mean age of 29.4 ± 9.6 years (range: 19 to 64 years), were enrolled in the study. The mean spherical equivalent refraction was −3.36 ± 2.46 diopters (D) (range: −8.875 to 1.375 D).

**Figure 1:**
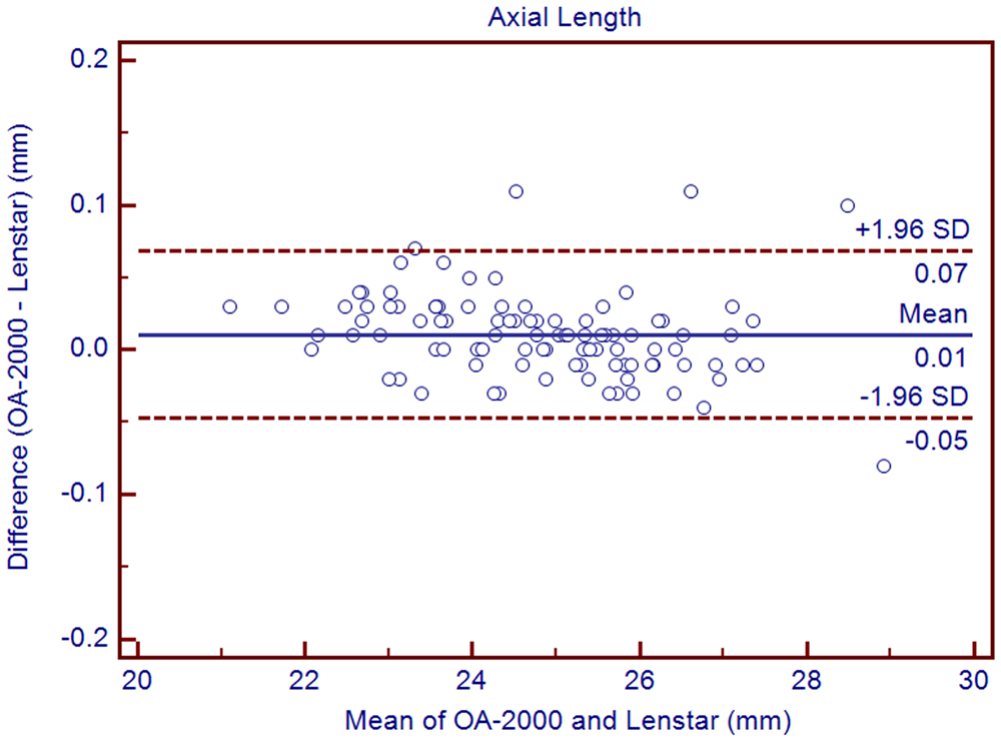
Bland-Altman plots show the agreement between OA-2000 swept-source optical coherence tomography and Lenstar optical low coherence reflectometry for measuring axial length. Solid lines represent the bias between both devices and dotted lines represent the 95% confidence interval for the difference.

**Figure 2:**
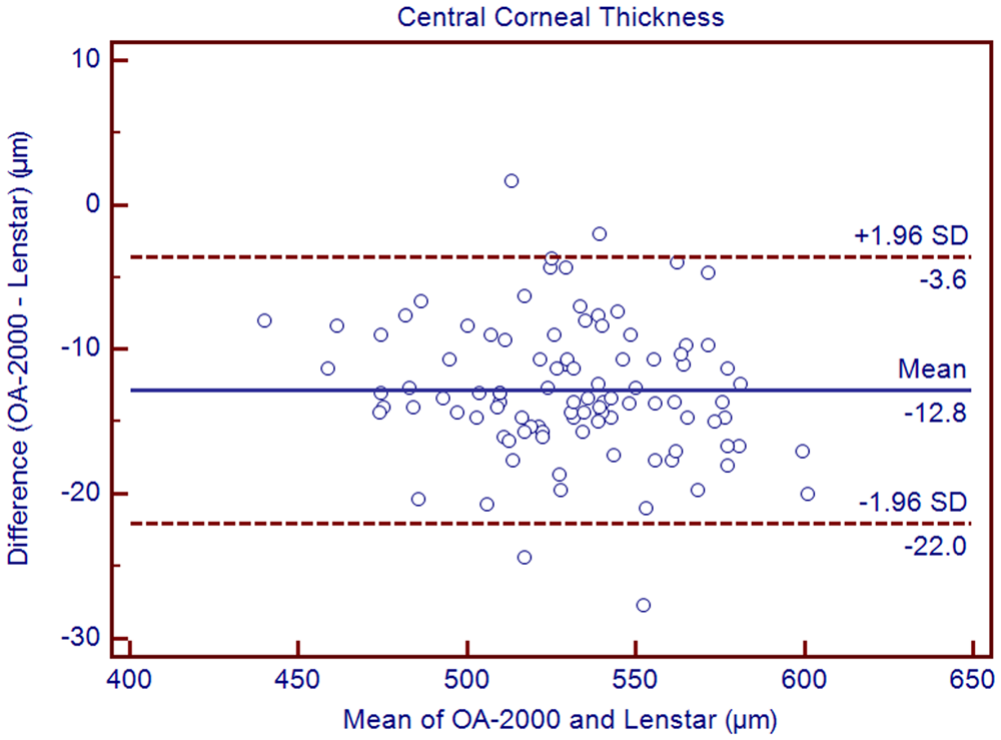
Bland-Altman plots show the agreement between OA-2000 swept-source optical coherence tomography and Lenstar optical low coherence reflectometry for measuring central corneal thickness. Solid lines represent the bias between both devices and dotted lines represent the 95% confidence interval for the difference.

**Figure 3:**
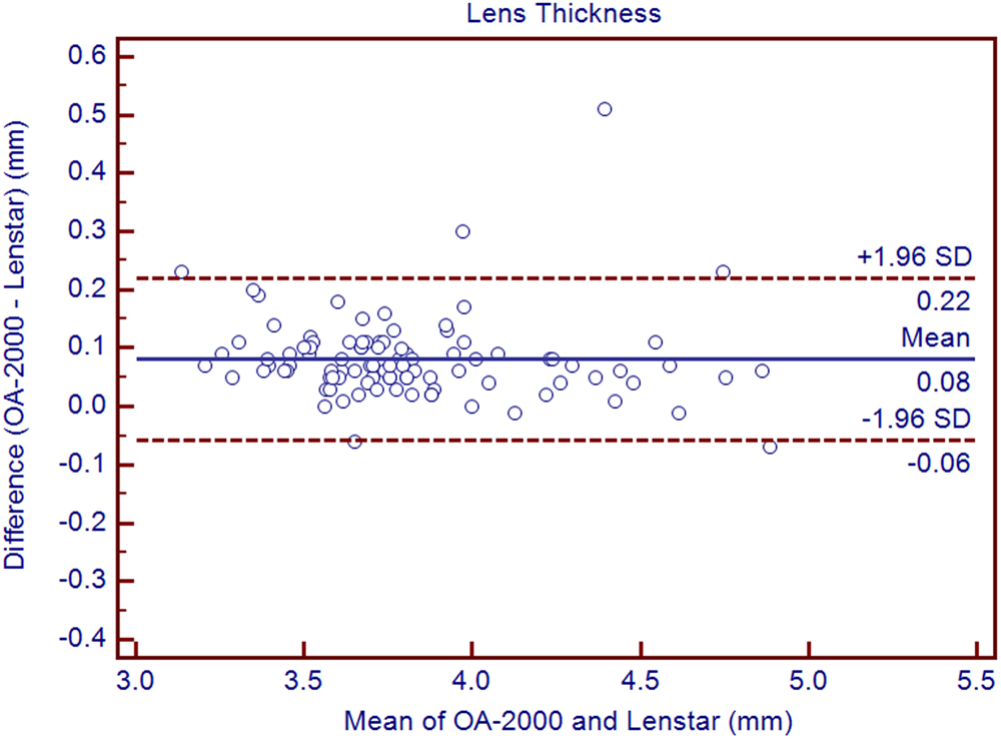
Bland-Altman plots show the agreement between OA-2000 swept-source optical coherence tomography and Lenstar optical low coherence reflectometry for measuring lens thickness. Solid lines represent the bias between both devices and dotted lines represent the 95% confidence interval for the difference.

**Figure 4:**
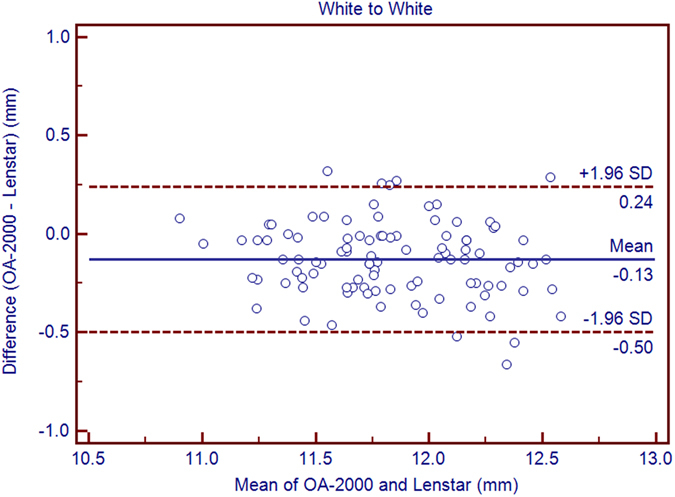
Bland-Altman plots show the agreement between OA-2000 swept-source optical coherence tomography and Lenstar optical low coherence reflectometry for measuring white-to-white. Solid lines represent the bias between both devices and dotted lines represent the 95% confidence interval for the difference.

**Figure 5:**
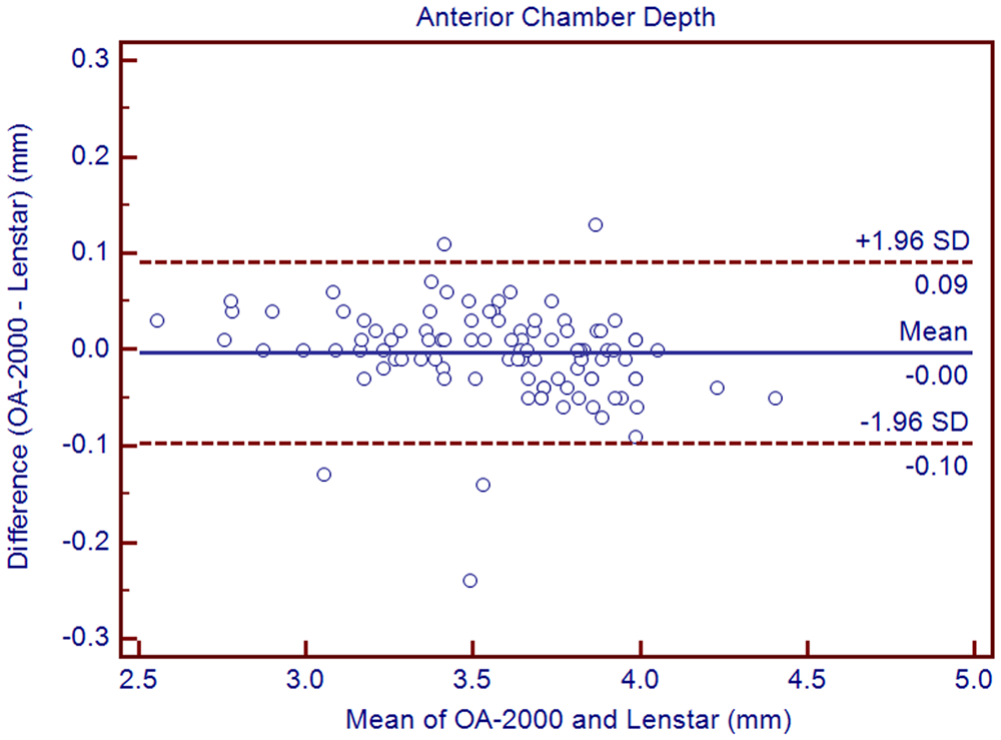
Bland-Altman plots show the agreement between OA-2000 swept-source optical coherence tomography and Lenstar optical low coherence reflectometry for measuring anterior chamber depth. Solid lines represent the bias between both devices and dotted lines represent the 95% confidence interval for the difference.

**Figure 6:**
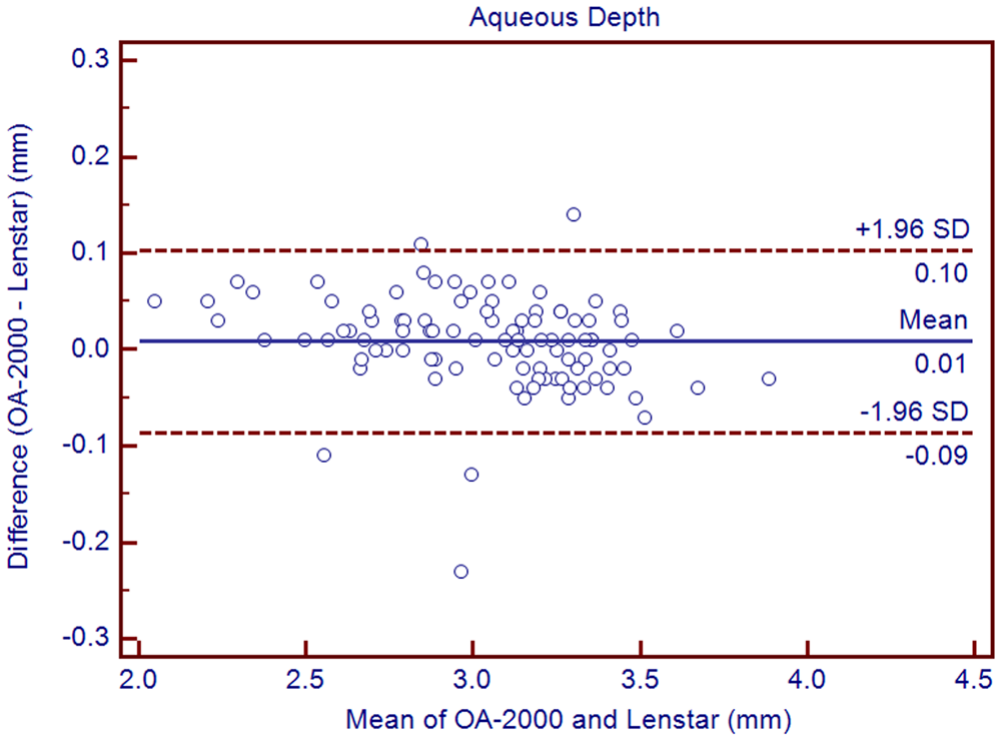
Bland-Altman plots show the agreement between OA-2000 swept-source optical coherence tomography and Lenstar optical low coherence reflectometry for measuring aqueous depth. Solid lines represent the bias between both devices and dotted lines represent the 95% confidence interval for the difference.

**Figure 7:**
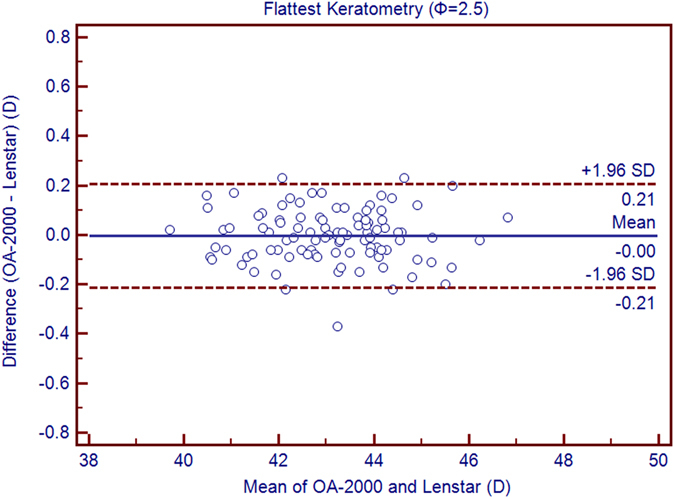
Bland-Altman plots show the agreement between OA-2000 swept-source optical coherence tomography and Lenstar optical low coherence reflectometry for measuring flat keratometry (Kf) at 2.5 mm. Solid lines represent the bias between both devices and dotted lines represent the 95% confidence interval for the difference.

**Figure 8:**
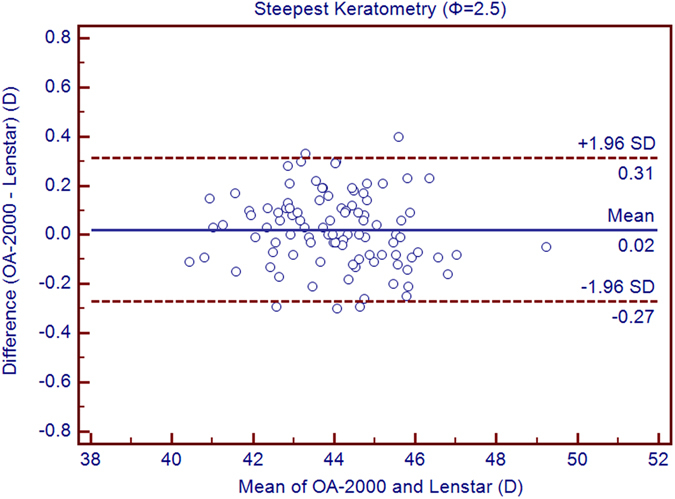
Bland-Altman plots show the agreement between OA-2000 swept-source optical coherence tomography and Lenstar optical low coherence reflectometry for measuring steep keratometry (Ks) at 2.5 mm. Solid lines represent the bias between both devices and dotted lines represent the 95% confidence interval for the difference.

**Figure 9:**
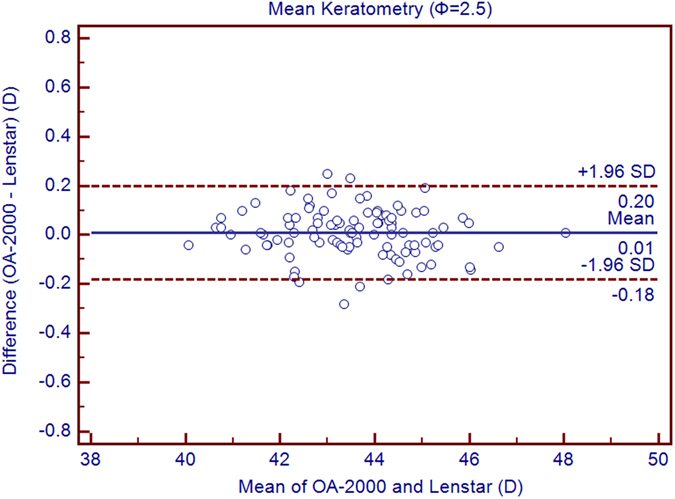
Bland-Altman plots show the agreement between OA-2000 swept-source optical coherence tomography and Lenstar optical low coherence reflectometry for measuring mean keratometry (Km) at 2.5 mm. Solid lines represent the bias between both devices and dotted lines represent the 95% confidence interval for the difference.

**Figure 10:**
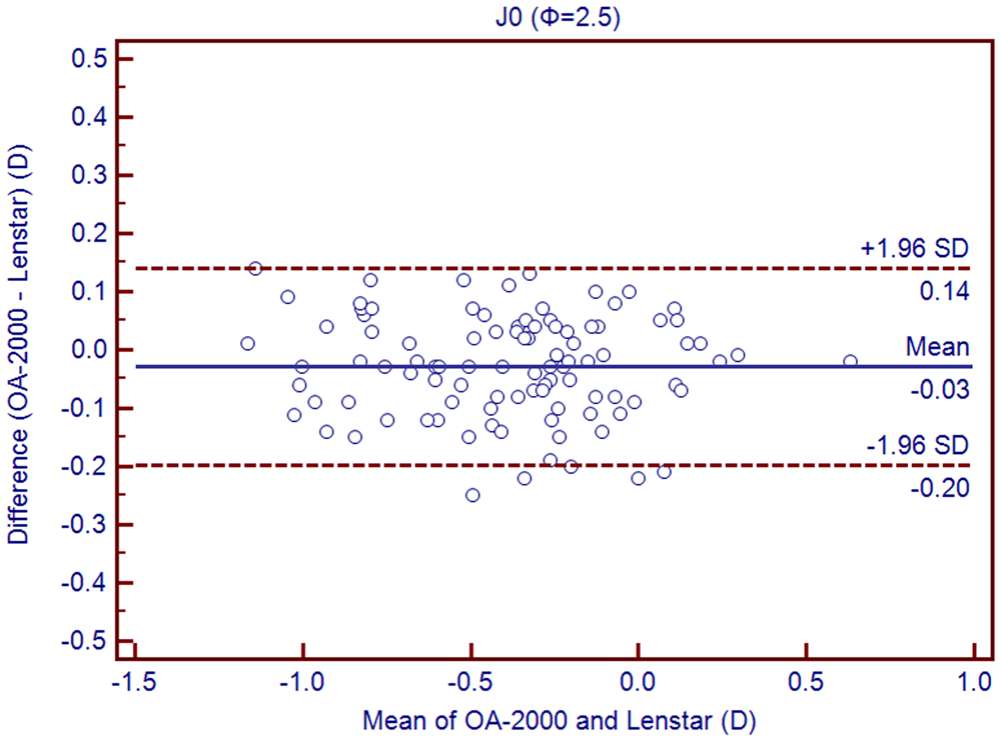
Bland-Altman plots show the agreement between OA-2000 swept-source optical coherence tomography and Lenstar optical low coherence reflectometry for measuring vectors J_0_ at 2.5 mm. Solid lines represent the bias between both devices and dotted lines represent the 95% confidence interval for the difference.

**Figure 11:**
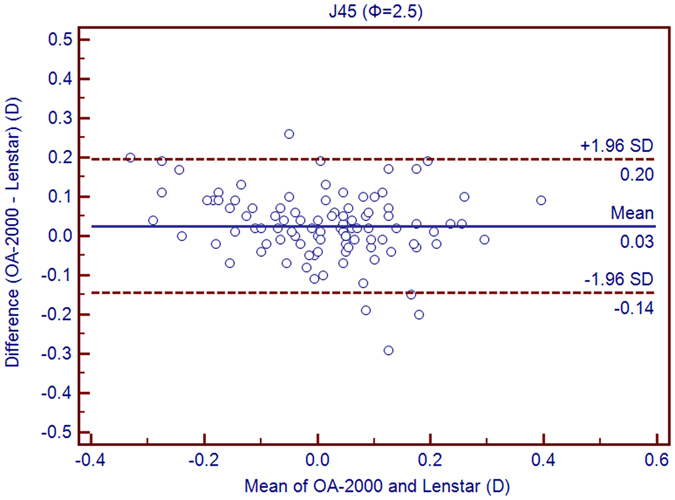
Bland-Altman plots show the agreement between OA-2000 swept-source optical coherence tomography and Lenstar optical low coherence reflectometry for measuring vectors J_45_ at 2.5 mm. Solid lines represent the bias between both devices and dotted lines represent the 95% confidence interval for the difference.

**Figure 12:**
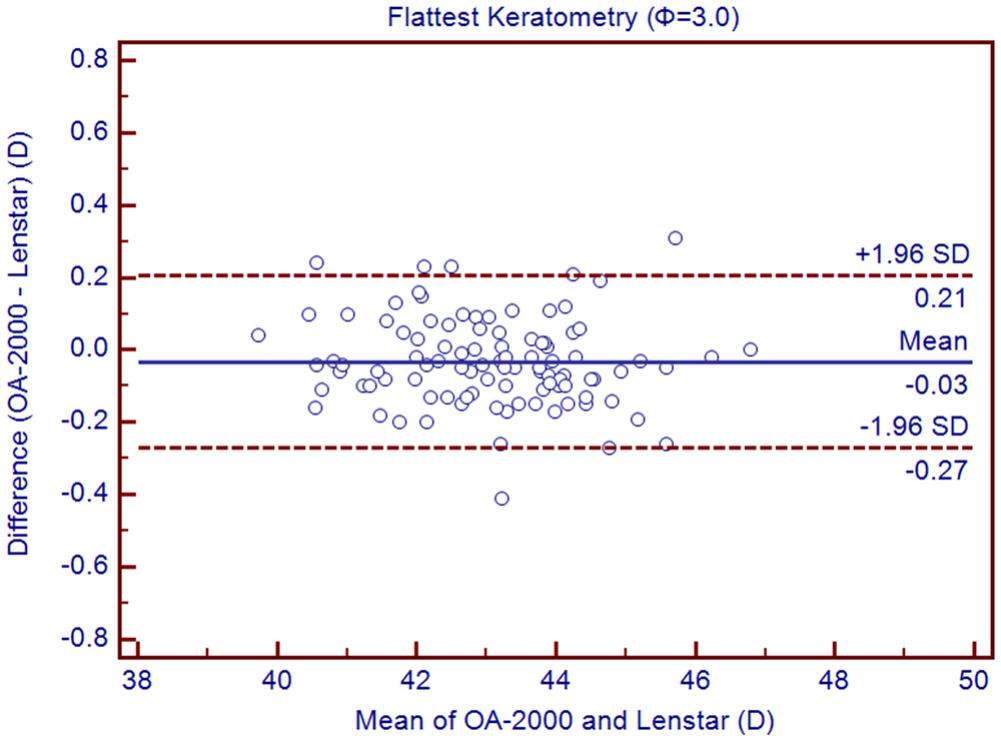
Bland-Altman plots show the agreement between OA-2000 swept-source optical coherence tomography and Lenstar optical low coherence reflectometry for measuring flat keratometry (Kf) at 3.0 mm. Solid lines represent the bias between both devices and dotted lines represent at the 95% confidence interval for the difference.

**Figure 13:**
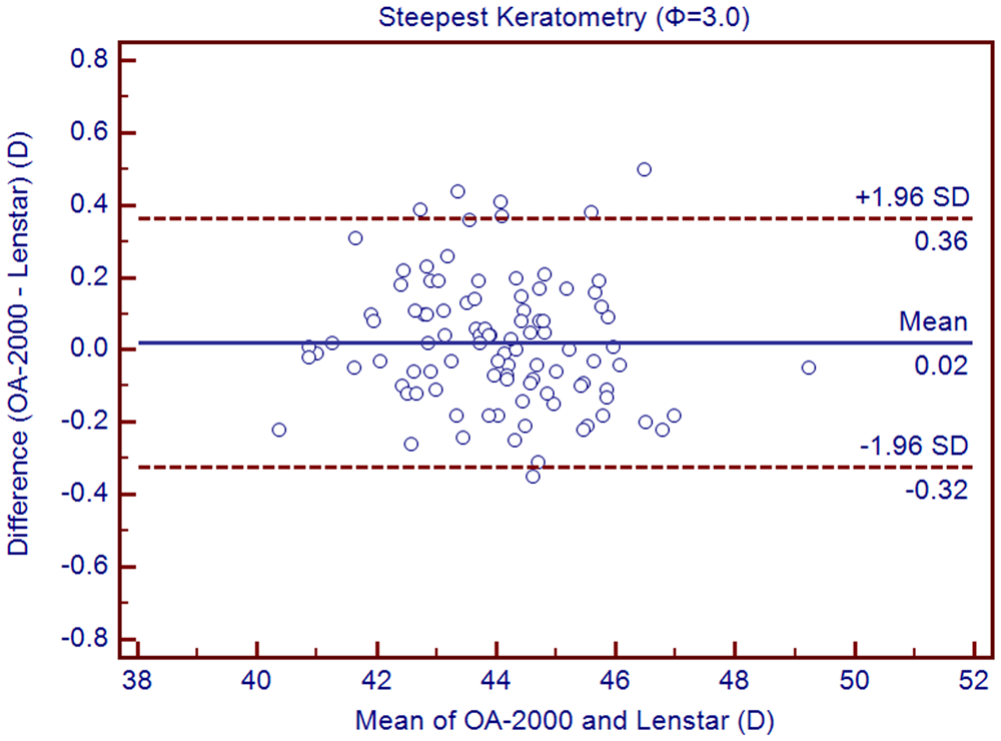
Bland-Altman plots show the agreement between OA-2000 swept-source optical coherence tomography and Lenstar optical low coherence reflectometry for measuring steep keratometry (Ks) at 3.0 mm. Solid lines represent the bias between both devices and dotted lines represent at the 95% confidence interval for the difference.

**Figure 14:**
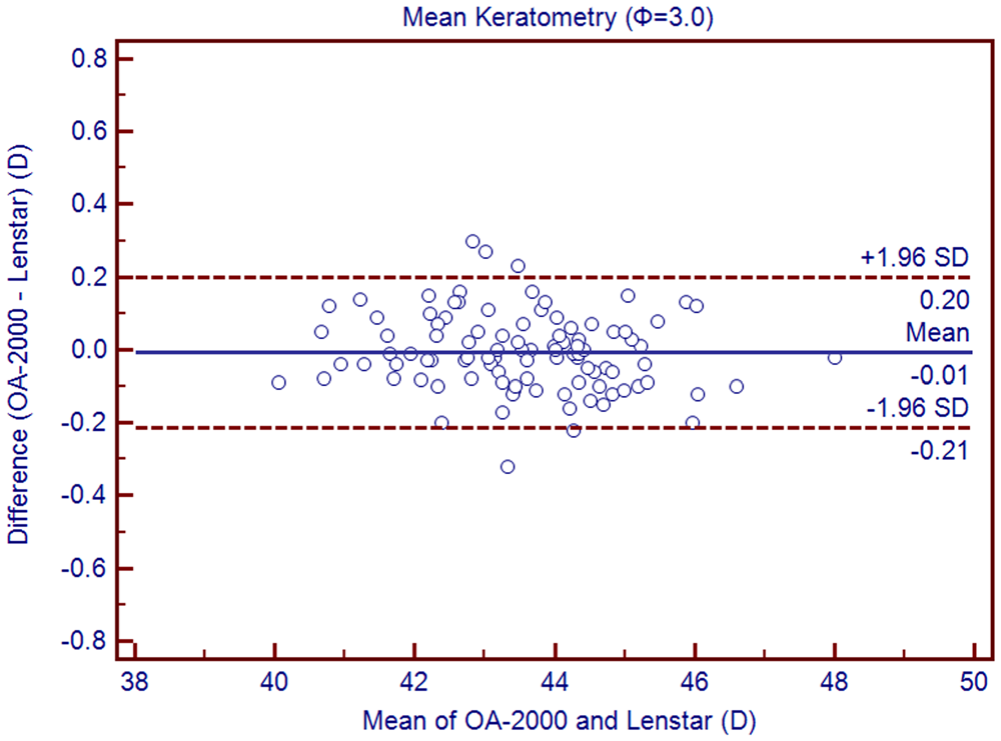
Bland-Altman plots show the agreement between OA-2000 swept-source optical coherence tomography and Lenstar optical low coherence reflectometry for measuring mean keratometry (Km) at 3.0 mm. Solid lines represent the bias between both devices and dotted lines represent at the 95% confidence interval for the difference.

**Figure 15:**
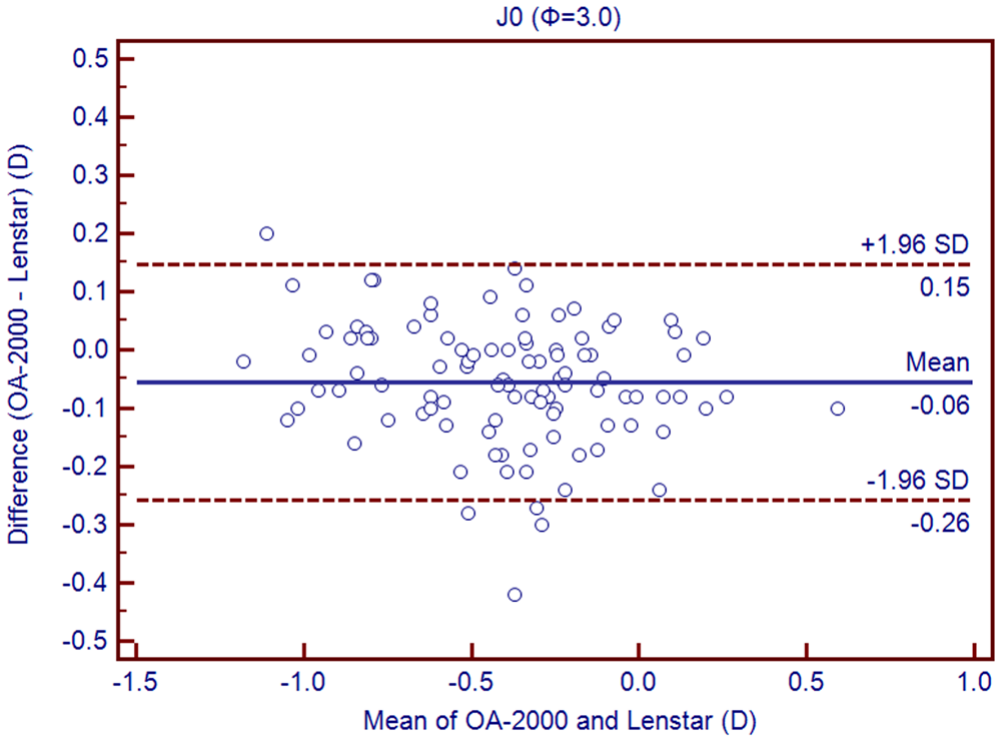
Bland-Altman plots show the agreement between OA-2000 swept-source optical coherence tomography and Lenstar optical low coherence reflectometry for measuring vectors J_0_ at 3.0mm. Solid lines represent the bias between both devices and dotted lines represent at the 95% confidence interval for the difference.

**Figure 16:**
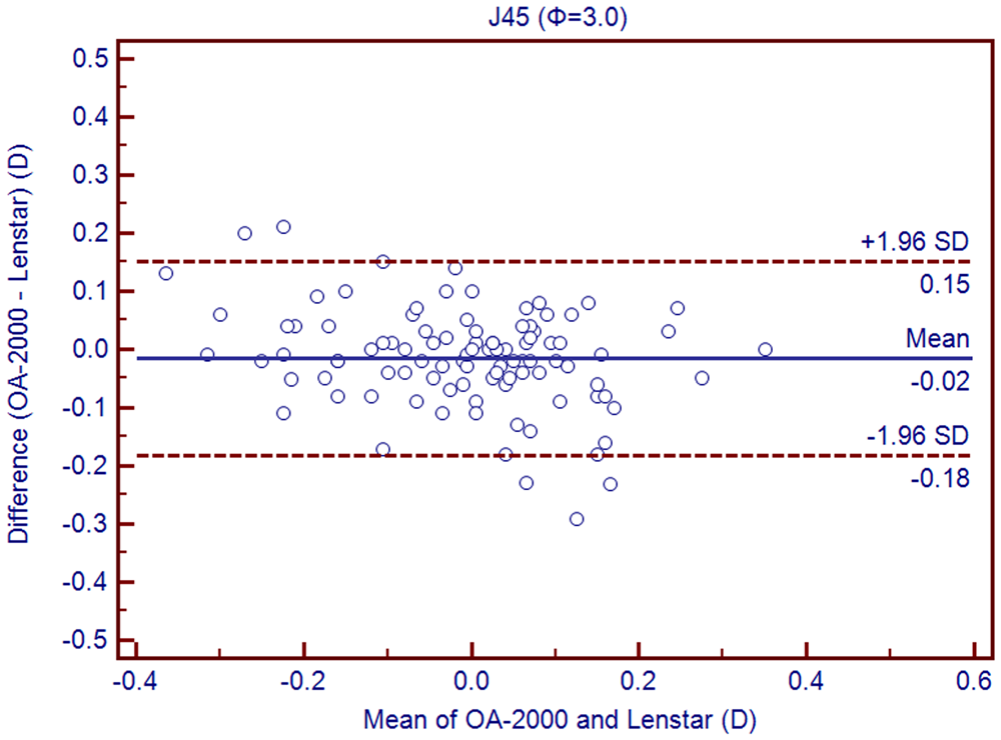
Bland-Altman plots show the agreement between OA-2000 swept-source optical coherence tomography and Lenstar optical low coherence reflectometry for measuring vectors J_45_ at 3.0 mm. Solid lines represent the bias between both devices and dotted lines represent at the 95% confidence interval for the difference.

Table 1 exhibits the mean, minimum and maximum values of the parameters obtained by the OA-2000 and Lenstar. The comparison and agreement data are reported in Table 2. The AL, CCT, LT and WTW values showed statistically significant differences (*P* < 0.01), but relatively narrow 95% LoA. The 95% LoA of AL was the narrowest with a range from −0.05 to 0.07 mm (Figs 1–4). The ACD and AD values by the two biometers were similar with a maximum absolute 95% LoA of 0.10 mm (*P* > 0.05) (Figs 5 and 6). The 95% LoA of corneal topography results including Kf, Ks, Km, J_0_ and J_45_ were in a narrow range with maximum values of −0.32 to 0.36 D, although some of them showed significant differences (*P* < 0.05) (Figs 7–16).

**Table 1:** The biometric measurement parameters provided by the OA-2000 swept-source optical coherence tomography and Lenstar optical low coherence reflectometry.

Parameter	OA-2000			Lenstar		
	Mean ± SD	Minimum	Maximum	Mean ± SD	Minimum	Maximum
AL (mm)	24.81 ± 1.51	21.12	28.87	24.80 ± 1.51	21.09	28.95
CCT (μm)	523.97 ± 31.59	436.00	591.00	536.80 ± 32.38	444.00	611.00
AD (mm)	3.04 ± 0.34	2.07	3.87	3.03 ± 0.35	2.02	3.90
ACD (mm)	3.57 ± 0.34	2.57	4.38	3.57 ± 0.35	2.54	4.43
LT (mm)	3.86 ± 0.37	3.24	4.89	3.78 ± 0.38	3.02	4.92
Kf (2.5 mm)	43.09 ± 1.39	39.72	46.86	43.09 ± 1.40	39.70	46.79
Ks (2.5 mm)	44.03 ± 1.49	40.37	49.20	44.01 ± 1.51	40.48	49.25
Km (2.5 mm)	43.56 ± 1.41	40.05	48.03	43.55 ± 1.43	40.09	48.02
AST (2.5 mm)	0.94 ± 0.57	0.10	2.34	0.92 ± 0.57	0.08	2.46
J_0_ (2.5 mm)	−0.40 ± 0.35	−1.16	0.62	−0.37 ± 0.36	−1.21	0.64
J_45_ (2.5 mm)	0.03 ± 0.13	−0.27	0.44	0.00 ± 0.15	−0.43	0.35
Kf (3.0 mm)	43.06 ± 1.38	39.74	46.79			
K2 (3.0 mm)	44.03 ± 1.49	40.26	49.20			
Km (3.0 mm)	43.54 ± 1.41	40.00	48.00			
AST (3.0 mm)	0.97 ± 0.55	0.14	2.41			
J_0_ (3.0 mm)	−0.43 ± 0.33	−1.19	0.54			
J_45_ (3.0 mm)	−0.02 ± 0.13	−0.32	0.35			
WTW (mm)	11.77 ± 0.38	10.94	12.68	11.90 ± 0.41	10.86	12.79

AL = Axial length, CCT = central corneal thickness, AD = aqueous depth, ACD = anterior chamber depth, LT = Lens thickness, Kf = flattest keratometry, Ks = steepest keratometry, Km = mean keratometry, WTW = white to white, SD = standard deviation.

**Table 2:** Agreement of biometric measurements between the OA-2000 swept-source optical coherence tomography and Lenstar optical low coherence reflectometry.

Device Pairings	Mean Difference ± SD	*P* Value	95% LoA
AL (mm)	0.01 ± 0.03	<0.001	−0.05 to 0.07
CCT (μm)	−12.83 ± 4.70	<0.001	−22.04 to −3.63
AD (mm)	0.01 ± 0.05	0.08	−0.09 to 0.10
ACD (mm)	0.00 ± 0.05	0.44	−0.10 to 0.09
LT (mm)	0.08 ± 0.07	<0.001	−0.06 to 0.22
Kf (2.5 mm)	0.00 ± 0.11	0.77	−0.21 to 0.21
Ks (2.5 mm)	0.02 ± 0.15	0.16	−0.27 to 0.31
Km (2.5 mm)	0.01 ± 0.10	0.38	−0.18 to 0.20
J_0_ (D)	−0.03 ± 0.09	<0.001	−0.20 to 0.14
J_45_ (D)	0.03 ± 0.09	<0.001	−0.14 to 0.20
Kf (3.0 mm)	−0.03 ± 0.12	0.01	−0.27 to 0.21
Ks (3.0 mm)	0.02 ± 0.17	0.26	−0.32 to 0.36
Km (3.0 mm)	−0.01 ± 0.11	0.56	−0.21 to 0.20
J_0_ (3.0 mm)	−0.06 ± 0.10	<0.001	-0.26 to 0.15
J_45_ (3.0 mm)	−0.02 ± 0.08	0.08	−0.18 to 0.15
WTW (mm)	−0.13 ± 0.19	<0.001	−0.50 to 0.24

AL = Axial length, CCT = central corneal thickness, AD = aqueous depth, ACD = anterior chamber depth, LT = Lens thickness, Kf = flattest keratometry, Ks = steepest keratometry, Km = mean keratometry, WTW = white to white, SD = standard deviation.

## Discussion

As a newly available optical biometer, the OA-2000 should be compared with the currently established biometers in clinical use^19^. The Lenstar was commercially available after the IOLMaster but provides more comprehensive parameters with different measuring principles, and its accuracy is well established^17^. An agreement analysis, based on the definitions adopted by the British Standards Institution as recommended by Bland and Altman, such as Bland-Altman plots and LoA, has been applied in our previous study but not been applied between the OA-2000 and Lenstar^20^. Thus, it is necessary to study the agreements and differences between the two devices, and detect and confirm the value and application prospect of the new device^21–23^. In the current study, according to our data, most parameters showed good agreement, especially for the AL, ACD and K values.

Minor difference was found between these two biometers for measuring AL, with a mean value of 0.01 mm. A 0.08 mm error in AL would result in an error of 0.20 to 0.34 D according to the SRK/T formula and a 0.10 mm measuring error may be equivalent to about 0.25 to 0.3 D in the spectacle plane. Thus, the maximum 95% LoA of 0.07 mm between OA-2000 and Lenstar was thought to be well acceptable in clinic^2, 17^. Our results were similar to those of Cruysberg *et al*. with a 95% LoA range of 0.07 to −0.01 mm between Lenstar and IOLMaster^17^. As compared to the results of Goebels *et al*. whose mean AL difference between the functional prototype and Lenstar in cataract, IOLMaster was −0.03 ± 0.09 mm and −0.02 ± 0.03 mm, respectively, mean difference was 0.03 mm with a large range of −0.48 to 0.67 mm, but our result was smaller and indicated excellent AL agreement^19^. Agreement in the current study was also better than that in Kongsap’s study, which reported a mean difference of −0.02 ± 0.03 mm between OA-2000 and IOLMaster 500 (Carl Zeiss Meditec, Dublin, CA, USA)^24^.

In the current study, the CCT provided by OA-2000 and Lenstar was 523.97 ± 31.59 μm and 536.80 ± 32.38 μm, respectively, the former also provided lower CCT values than Lenstar in a previous study^19^. This slight difference may be due to the different algorithms and analysis programs of the two devices for boundary determination. A comparison between IOLMaster 700 (Carl Zeiss Meditec AG), which adopted SS-OCT technology and Lenstar showed good agreement with 95% LoA of −8.69 to 8.99 μm^25^. The Lenstar also showed good agreement with the ultrasound (95% LoA was −6.8 to 9.6 μm in unoperated eyes) and RTVue OCT (RTVue-100; Optovue Inc., Fremont, CA, USA) (95% LoA was −3.1 to 11.8 μm), which were a little narrower than our results^26, 27^. But the CCT values showed a relatively similar range of 95% LoA between Lenstar and Visante AS-OCT (Carl Zeiss Meditec, Dublin, California, USA) in the study by Cruysberg *et al*. with a maximum value of 24.1 μm, which was slightly larger than in our study^17^. Similar results were found in the comparison between Sirius (Costruzione Strumenti Oftalmici, Florence, Italy) and ultrasound, which also showed good agreement for CCT measurements^28^. When the CCT values were compared between SP-3000P (NCSM; Topcon Corp, Tokyo, Japan), EM-3000 (Tomey, Nagoya, Japan), SP-02 (Costruzione Strumenti Oftalmici, Italy) and ultrasound pachymetry (Tomey Inc., Nagoya, Japan), the 95% LoA ranges were broader than ours (the minimum range was −29.3 to 1.7 for comparison between SP-3000P and EM-3000, the maximum range was −62.1 to 8.7 for comparison between SP-3000P and SP-02)^29^. As the CCT vary up to 20 μm over a day in individuals and every 25 μm deviation would lead to about 1 mmHg correction, the current result was clinically acceptable and showed moderate agreement^25, 30^.

The ACD and LT values may be affected by accommodation, but the former only changed by −0.09 to −0.14 mm with accommodation in myopia eyes^31–33^. The ACD did not show significant difference with accommodation in emmetropic subjects in the study by Dominguez-Vicent *et al*.^34, 35^. The OA-2000 and Lenstar use similar light spot for focusing and automatically measure along the visual axis, which reduces the accommodation influence. The differences between them for AD, ACD and LT values were low to 0.01 ± 0.05 mm, 0.00 ± 0.05 mm, 0.08 ± 0.07 mm within a clinically acceptable narrow 95% LoA range in the current study. Agreement was higher with respect to comparisons among the functional prototype of OA-2000 and Lenstar, IOLMaster 500, and AL-Scan (Nidek Co, Aichi, Japan) in previous studies^19, 24, 36^. It is surprising to compare agreement between OA-2000 and Lenstar for ACD measurements in the present study and in the study by Goebel *et al*.: in our sample the mean value was the same for both devices (3.57 mm), whereas in the above-mentioned paper the OA-2000 provided a remarkably higher mean value (3.71 vs 3.09 mm). It is likely that such a discrepancy depends on the fact that Goebel *et al*. investigated a functional prototype based on OLCR, while we analyzed the commercially available machine based on SS-OCT. Technical development of the OA-2000 from the prototype to the final version may explain these differences. The good performance and agreement suggested a potential application in the IOL power formulae of new generation, which use ACD and LT as additional variables^2, 37^.

In this study, the K values from OA-2000 were simultaneously assessed at 2.5 mm and 3.0 mm diameters, while central corneal topography provided by the Lenstar was at 1.65 mm and 2.3 mm. As J_0_ and J_45_ based on Fourier transformation were shown to be useful in previous studies for astigmatism evaluation, we additionally calculated these results^38^. All the results showed good agreement with a maximum 95% LoA of 0.36D although the differences of K values at 3.0 mm were slightly larger than those at 2.5 mm, but relatively better than that between AL-Scan and Lenstar^36^. The total measurement errors of Lenstar were 0.18D and 0.24D for anterior steep and flat keratometry, respectively, and they were close to that of Placido^39^. Comparison of keratometry obtained by Lenstar and Topolyzer (WaveLight Technologie AG, Erlangen, Germany), Galilei G6 (Ziemer, Port, Switzerland) showed acceptable 95% LoA range but not as narrow as that in our current study^40, 41^. Although Goebels *et al*. had shown the R1 and R2 values of OA-2000 and Lenstar, they were mainly comparing the AL and ACD. This was the first time that the differences of keratometry between OA-2000 and Lenstar were comprehensively analyzed, and good agreements were achieved^19^.

The agreement of WTW measurement was not always good in prior studies because of the edge recognition technology. The Lenstar showed good agreement with IOLMaster in natural pupils but not in cycloplegic subjects^42^. The measurements were also similar between Lenstar with AL-Scan but not with Sirius^36, 43^. Currently, better agreement between OA-2000 and Lenstar than that between AL-Scan and Lenstar (95% LoA ranged from −0.69 to 0.34 mm), the OA-2000 and IOLMaster 500 (95% LoA ranged from −1.85 to 1.42 mm) was found with narrower 95% LoA range of −0.50 to 0.24 mm^24, 36^.

There were some limitations in this study. We only included healthy volunteers, and their ocular condition was easily captured due to their good cooperation and transparent refractive media. We confirmed that the OA-2000 has a significant advantage when measuring AL in cataract patients with respect to the IOLMaster and Aladdin optical biometer (Topcon, Japan)^44^. But the success rate of OA-2000 in patients with other ocular diseases still needs further analysis. Besides, as a type of SS biometer, comparisons with other SS-OCT should be performed in the future.

In summary, most parameters acquired by OA-2000 were comparable to those obtained by Lenstar including AL, CCT, AD, ACD, LT, K values and WTW, with relatively narrow 95% LoA range. The AL values showed the best agreement among the available parameters.

### Subjects and Methods

This prospective study recruited 99 normal subjects from the Eye Hospital of Wenzhou Medical University. The research protocol was in accordance with the tenets of the Declaration of Helsinki, and was approved by the Office of Research Ethics, Eye Hospital of Wenzhou Medical University. All subjects signed informed consent after understanding the purpose of the research.

The exclusion criteria were recent contact lens wear (rigid contact lens within four weeks and soft contact lens within two weeks), any active ocular pathology, history of ocular surgery and trauma, intraocular pressure >21 mmHg, fundus disease and systemic diseases with ocular symptoms.

The Lenstar applies an OLCR principle to measure AL, CCT, ACD, and LT with the wavelength of 820 nm superluminescent diode laser. It also employs 950 nm light to measure WTW, pupil diameter (PD), corneal curvature and pupil by image analysis. The flattest meridian K and steepest meridian K values are analyzed by the position of 32 projected light reflections at two rings with diameters of 1.65 and 2.30 mm. Keratometry is calculated by transforming the corneal curvature radius into diopters (D) using the 1.3375 refractive index. WTW is measured using the image of the corneal radius and the iris derived from keratometry.

The OA-2000 uses the Placido ring cone and optical interference principle to measure the axial dimensions of the ocular structure. The Fourier domain optical interference is applied to measure the AL, CCT, ACD and LT parameters using the wavelength of 1060 nm swept source laser. The 515 nm and 750 nm light-emitting diodes (LEDs) are used to determine the K values, WTW and PD, respectively. It also employs the Placido ring cone to simultaneously measure corneal shape and the radius of corneal curvature at 2.5 mm and 3.0 mm positions. Each Placido ring is 256 points. It totally captures 2,304 points by nine rings.

We analyzed AD and ACD, respectively. The former is the distance from the corneal endothelium to the anterior surface of the lens capsule, while the latter represents the distance from the corneal epithelium to the lens^45^. Astigmatism was analyzed using J_0_ and J_45_ vectors according to the following formulae^38^:

$$\begin{array}{c}{{\rm{J}}}_{0}=(-\mathrm{cylinder}/2)\,\cos \,(2\times {\rm{axis}})\\ {{\rm{J}}}_{45}=(-\mathrm{cylinder}/2)\,\sin \,(2\times {\rm{axis}})\end{array}$$

All subjects were requested to sit with their foreheads against the headrest, chins on a chin-rest, open both eyes and fixate on an internal target within each device. They were instructed to completely blink twice in order to spread an optically smooth tear film on the cornea before each measurement. After each scan, the device was moved backward and realigned for the next measurement. Each subject was measured until three valid results were obtained by each device. The sequence order measurement of the Lenstar and the OA-2000 was confirmed by the MedCalc Statistical Software (version 13.0, MedCalc Software Inc., Belgium). In order to avoid the effects of diurnal variation in corneal shape and ocular structure, the entire examination was accomplished in less than 15 minutes^46^. Only one eye of each subject was chosen to be measured in order to avoid structural similarities between fellow eyes^47^.

### Statistical Analysis

All data were analyzed using SPSS software for Windows version 21 (IBM corporation, USA), MedCalc statistical software (version 14.1, MedCalc Software Inc, Belgium) and Microsoft Office Excel 2010 (Microsoft Corp, WA, USA). *P* < 0.05 was considered as statistically significant. Kolmogorov-Smirnov test was applied to confirm that the data were normally distributed. The results were expressed as the means ± standard deviations (SD).

Paired *t-*tests were used to compare the differences in ocular biometry measurements (AL, ACD, AD, CCT, LT, WTW and keratometry) between the Lenstar and the OA-2000. Bland-Altman plots were used to assess the agreement between the two devices. The 95% limits of agreement (LoA) were expressed as the mean difference ± 1.96 SD of difference, which indicated an interval within which 95% of the differences between measurements are expected to lie^21, 48^.
